# Promote a new paradigm to prevent neurodegenerative disease in sub-Saharan Africa

**DOI:** 10.11604/pamj.2020.36.138.21881

**Published:** 2020-06-29

**Authors:** Callixte Kuate-Tegueu, Pierre Waffo-Teguo, Nadine Simo, Maturin Tabue-Teguo

**Affiliations:** 1 Faculty of Medicine and Biomedical Sciences, University of Yaounde I, Yaounde, Cameroon,; 2 Institut des Sciences de la Vigne et du Vin, EA 4577, USC1366 INRA, IPB, Université de Bordeaux, Bordeaux, France,; 3 Université de Guadeloupe, Centre Hospitalier Universitaire de Pointe-à-Pitre, Les Abymes, France,; 4 INSERM U 1219, Université de Bordeaux, Bordeaux, France

**Keywords:** Cohort study, epidemiology, neurodegenerative disease, sub-Saharan-Africa (SSA)

## To the editors of the Pan African Medical Journal

Prevention is the corner stone when dealing with disability as it must be considered as a nearly irreversible condition in elderly people. There is a growing demand for the identification of effective solutions against the detrimental consequences that age-related conditions (in particular, disabilities) exert on our healthcare systems. Therefore, preventive interventions able to modify the natural history of age-related conditions are urgently needed. Nutritional interventions appear to be a potentially effective way to prevent dementia in high income countries. The promotion of a healthy lifestyle and the adoption of preventive countermeasures for a specific condition are quite challenging. The approach based on the use of local natural resources such as diversified and rich food must be explored in sub-Saharan-Africa (SSA).

**Dementia: a global public health priority:** as demographics in developed nations shift towards an aging population, neurodegenerative pathologies pose one of the largest challenges to the modern health care system [1]. Neurodegenerative diseases are a growing health concern. The increasing incidences of these disorders have a great impact on the patients' quality of life [2]. The most common neurodegenerative diseases are Alzheimer´s disease and Parkinson´s disease, but also include other conditions like amyotrophic lateral sclerosis and others dementia. These conditions are often debilitating, not just for the individual diagnosed but for his or her entire family, social network and health care system. To this project, we are focused on Alzheimer´s disease as neurodegenerative diseases. Alzheimer´s disease (AD) is a progressive neurodegenerative disorder that accounts for the major cause of dementia in the world. The number of cases is projected to reach 106.8 million worldwide by the year 2050. Therefore, the disease is a growing public health concern with major socioeconomic burden [2]. Much attention has been paid to disease-modifying factors and risk factors for AD [3]. Little is known about the natural history, clinical pattern, etiologies and treatment status of AD in elderly people in sub-Saharan-Africa. In this region, data about the prevalence of neurodegenerative pathologies are scarce and largely coming from Central and Western Africa [4-6]. The reported age-adjusted prevalence of dementia for the population-based studies in SSA varied widely, ranging from 2.3% to 7.6%. Many risk factors of neurodegenerative pathologies have been highlighted, leaving a door open towards prevention.

**Importance of prevention for neurodegenerative disease:** prevention is the corner stone when dealing with disability as it must be considered as a nearly irreversible condition in elderly people. There is a growing demand for the identification of effective solutions against the detrimental consequences that age-related conditions (in particular, disabilities) exert on our healthcare systems. Therefore, preventive interventions able to modify the natural history of age-related conditions are urgently needed. In this last decade, a relevant body of scientific literature has increasingly been advocated the need of implementing preventive actions against age-related and disabling conditions in the elderly in high income countries. Current treatments given after dementia diagnosis have demonstrated only modest efficacy. Taking into account the failure of these curative therapies, it seems reasonable to evaluate another strategy in patient´s management such as possibility of preventive intervention that could reduce the rate of conversion to dementia among subjects at high risk [7-9]. Nutritional interventions appear to be a potentially effective way to prevent dementia [10] in high income countries.

**Suggestions to implement neurodegenerative disease prevention interventions in SSA:** to our knowledge, no epidemiological study nor clinical trial has yet reported the implementation prevention intervention against neurodegenerative disease in SSA, which still is a vast African region with a population of 1.1 billion people and a steadily increasing number of elders (projected to be over 67 million by 2030 [11]). The allocation of economic resources in the field of prevention (for any kind of legitimate clinical condition) may raise special issues, especially in SSA. In these regions where economic difficulties may already affect the condition of optimal healthcare services, the development of novel infrastructures dedicated to preventive medicine might be difficult. Thus, the legislator may find himself at balancing the costs of prevention with those necessary for assuring the sustainability of the traditional clinical care services. Such decision may potentially foster ethical discussions. The activities aimed at informing the general population about the risk of disabling conditions at old age are necessary but inevitably expensive, challenging their feasibility especially in SSA. Moreover, the promotion of a healthy lifestyle and the adoption of preventive countermeasures for a specific condition are quite challenging. For this reasons, an approach based on the use of local natural resources such as diversified and rich food must be explored in SSA. One of the candidate is *Gnetum africanum*, popular food plants in tropical Africa. *Gnetum africanum* is a liana that grows abundantly in Central Africa. Leaves are eaten as raw vegetables or in soups by local people. It is known as “Eru or Kok” in Cameroon. The leaves are also used for their nutritional and medicinal properties [12]. *G. africanum*is a rich source of health promoting compounds, including flavonoids, phenolic acids, and stilbenoids [12-14]. The stilbenoid monomer resveratrol has undergone extensive biological testing, particularly in regard to disease prevention and anti-aging activities [15-17]. Cameroon has launched a program to support the cultivation of *G. africanum*, which has produced more than 3 million plants and trained more than five million people. We plan a study whose aim will be to determine the relationship between sociodemographic factors, *G. africanum* intake, and the presence of neurodegenerative disease and secondary frailty in people from Cameroon, a Central African country. The result of this study may lead to a possible preventive effect of African antioxidants rich foods by reducing cognitive decline. In other hand, the identification of frail older persons is a public health priority. Frailty is defined as an extreme vulnerability of the organism to endogenous and exogenous stressors, a syndrome that exposes the individual at higher risk of negative health-related outcomes as well as a transition phase between successful aging and disability. The theoretical concept of frailty is largely agreed, its practical translation still presents some limitations due to the existence of multiple tools and operational definition Theses interventions should be developed against frailty and the resulting effects of ageing [11].
